# TSS-Captur: a user-friendly pipeline for characterizing unclassified RNA transcripts

**DOI:** 10.1093/nargab/lqae168

**Published:** 2024-12-18

**Authors:** Mathias Witte Paz, Thomas Vogel, Kay Nieselt

**Affiliations:** Institute for Bioinformatics and Medical Informatics, Department of Computer Science, University of Tübingen, Sand 14, Tübingen 72076, Germany; Institute for Bioinformatics and Medical Informatics, Department of Computer Science, University of Tübingen, Sand 14, Tübingen 72076, Germany; Institute for Bioinformatics and Medical Informatics, Department of Computer Science, University of Tübingen, Sand 14, Tübingen 72076, Germany

## Abstract

RNA-seq and its 5′-enrichment methods for prokaryotes have enabled the precise identification of transcription start sites (TSSs), improving gene expression analysis. Computational methods are applied to these data to identify TSSs and classify them based on proximal annotated genes. While some TSSs cannot be classified at all (orphan TSSs), other TSSs are found on the reverse strand of known genes (antisense TSSs) but are not associated with the direct transcription of any known gene. Here, we introduce TSS-Captur, a novel pipeline, which uses computational approaches to characterize genomic regions starting from experimentally confirmed but unclassified TSSs. By analyzing TSS data, TSS-Captur characterizes unclassified signals, complementing prokaryotic genome annotation tools. TSS-Captur categorizes extracted transcripts as either messenger RNA for genes with coding potential or non-coding RNA (ncRNA) for non-translated genes. Additionally, it predicts the transcription termination site for each putative transcript. For ncRNA genes, the secondary structure is computed. Moreover, all putative promoter regions are analyzed to identify enriched motifs. An interactive report allows seamless data exploration. We validated TSS-Captur with a *Campylobacter jejuni* dataset and characterized unlabeled ncRNAs in *Streptomyces coelicolor*. TSS-Captur is available both as a web-application and as a command-line tool.

## Introduction

In molecular biology and genetics, the identification of transcription start sites (TSSs) can provide valuable insights into the regulation of gene expression in prokaryotic organisms. For example, it can aid researchers to understand the mechanisms controlling transcription initiation by allowing the identification of nearby transcription factor binding sites (1) and hence provide new insights into gene regulation. Differential RNA-seq (dRNA-seq) (2) and Cappable-seq (3) are enrichment methods for RNA-seq that allow the identification of TSSs in prokaryotes. These methods modify the RNA libraries prior to sequencing, allowing for the base-specific identification of the 5′-end of expressed genes. Such experiments typically result in hundreds to thousands of TSSs, making a manual determination cumbersome. To facilitate the process of TSS identification, computational methods have been developed in the past years.

TSSpredator (4) is one such tool that determines TSS signals from experimental data. It then associates the identified TSSs with annotated genes based on their proximity and strand. Studies that have generated genome-wide TSS maps across various bacterial genomes have led to important insights into gene regulation and expression. One study, a genome-wide transcriptome analysis of *Helicobacter pylori*, uncovered regulatory mechanisms within the 5′-untranslated regions (5′-UTR) of annotated genes (5). A more recent study reported a genome-wide TSS map for *Bacteroides thetaiotaomicron* (6). Besides providing insight into the vast transcription regulation mechanisms of the organism, this study reported around 269 potential non-coding RNA (ncRNA) candidates, including *GibS*, a *trans*-acting RNA gene involved in bacterial metabolism regulation. In these and many other studies, numerous TSS signals were not found close to any annotated gene. Hence, such TSSs were typically reported as orphan TSSs (oTSSs). Moreover, other TSSs were located on the antisense strand within the coordinates of an annotated gene (thus typically called antisense TSSs [aTSSs]) but have no annotated gene in the direct downstream region.

Genome annotation pipelines, such as Bakta (7), Prokka (8) and the Prokaryotic Genome Annotation Pipeline (PGAP) (9), primarily focus on identifying sequences with coding potential, located within messenger RNA (mRNA) transcripts, as well as housekeeping ncRNA genes. Other ncRNA genes are often overlooked due to their diverse structural patterns. In addition, the complexity also increases, since ncRNA genes with low sequence similarity may fold into the same or very similar structures. This structural and sequence heterogeneity of ncRNA genes makes their computational prediction challenging, especially in prokaryotes (8,10,11). Nevertheless, prokaryotic ncRNA genes play important functional roles, such as the *cis* or *trans* action of small RNAs (sRNAs) (12). Orphan and aTSSs are promising signals that may indicate the presence of overlooked genes, such as these ncRNAs. Moreover, regions starting at these sites may also transcribe previously unidentified protein-coding genes, such as small open reading frames (sORFs), which have proven equally challenging to characterize (13,14). Thus, an exploratory tool designed to automatically characterize the transcripts starting at these sites as well as their respective upstream regions would offer valuable insights into the prokaryotic transcriptome.

To enable the characterization of these TSS classes and their downstream regions in an explorative manner, we introduce TSS-Captur, a TSS-characterization pipeline for unclassified RNA-transcripts. TSS-Captur combines the results of transcriptomic experiments with the underlying genomic sequence, allowing the characterization of putative transcripts, starting with orphan and aTSS signals predicted using TSSpredator. For the overall characterization, TSS-Captur integrates different tools. TSS-Captur determines whether a transcript beginning at an oTSS or aTSS transcribes into a mRNA (protein-coding) or into a ncRNA (non-coding gene). For this, it combines two well-established prediction methods: a comparative genomics approach (QRNA (15)) and an *ab initio* method (Coding-Non-Coding Identifying Tool, in short CNIT (16)). Furthermore, TSS-Captur associates TSS signals with predicted transcription termination sites (TTSs) to compute the length of the transcript. It also provides the visualization of the computed secondary structure of ncRNA genes, enabling the user to explore the thermodynamic characteristics (i.e., folding potential) of such genes. Lastly, TSS-Captur facilitates the analysis of motifs within the regions upstream of each TSS signal for the identification of transcription factor binding sites or other regulatory elements. All these results are presented in an interactive report that offers both an overview of the predicted results and detailed information for each transcript. Implemented as a stand-alone command tool and as an interactive web tool, TSS-Captur offers a user-friendly complement to existing annotation methods, aiming to enhance understanding of the bacterial transcriptome.

## Related work

The accuracy and completeness of a genome annotation is a key part for multiple computational analyses, since it provides a baseline for the functional potential of an organism (7). Typical prokaryotic genome annotation pipelines, such as Bakta, Prokka or PGAP, not only lack the annotation of 5′- and 3′-UTRs but also of small ncRNAs (7–9). Other approaches, such as Promotech (17) or G4promfinder (18), complement these predictions by characterizing promoter regions. However, the diverse regulatory mechanisms in prokaryotes present challenges in generalizing these methods. Adding the transcriptomic layer of information can provide more precise or even potentially new results, such as refining a transcript’s structure or identifying previously unannotated expressed regions. Tools such as APERO (19) and baerhunter (20) integrate transcriptomic data with genome data by using mapped RNA-seq reads to identify expressed regions that are not proximal to annotated genes, thereby identifying potential small non-coding genes. However, RNA-seq data alone have been proven to be insufficient for an accurate base-exact prediction of gene boundaries (5). Instead of relying on RNA-seq data alone, ANNOgesic (21) uses dRNA-seq data to identify TSSs and their corresponding transcripts. ANNOgesic offers a plethora of different methods for enhancing bacterial genome annotations, including the prediction of sRNAs, sORF, circular RNAs, riboswitches, RNA thermometers and RNAs related to Clustered Regularly Interspaced Short Palindromic Repeats (CRISPR). However, ANNOgesic lacks a user-friendly interface for executing and exploring results, making it difficult to confirm and further investigate its predictions. Our approach, TSS-Captur, complements ANNOgesic by providing an intuitive web-based platform for exploration.

## The TSS-Captur pipeline

TSS-Captur is a pipeline that characterizes genomic regions starting with TSS signals that cannot be associated with any known annotated gene (orphan or aTSSs). It is based on Nextflow DSL2 (22) for the stage management and Docker for the containerization of all required tools. To facilitate the user experience, TSS-Captur can be used as a web application (Supplementary Figure S1), and results can be explored through an interactive web interface (Supplementary Figure S2). The web-application is based on NextJS, while the report is created by Frozen-Flask and the JavaScript library DataTables.

**Figure 1.**
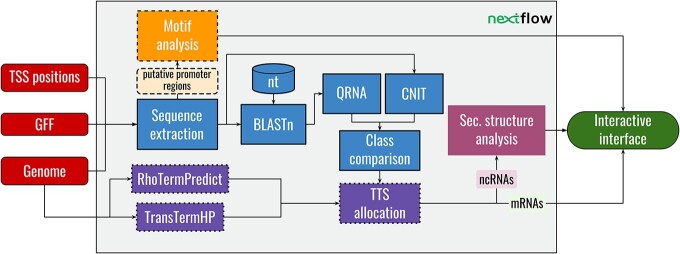
Overview of TSS-Captur. Different shapes have been used to represent data (ovals), processes (rectangles) or databases (cylinders). The input files (left-most ovals) are provided to Nextflow for the data preparation. This provides the input to the RNA classification (solid lines) and the motif analysis (dashed lines). The transcription termination site (TTS) prediction (dotted lines) works directly on the genome and the results are combined with the classified transcripts. Here, transcripts classified as ncRNAs are analyzed for their secondary structure (no borders). Lastly, all results are presented in the interactive interface (right-most oval).

We also provide a stand-alone command-line version of the software. In the ensuing sections, we provide a short overview of the TSS-Captur pipeline (see Figure 1), followed by detailed explanations of each step.

TSS-Captur requires three different input files: the MasterTable from the TSS determination and classification process run by TSSpredator, as well as the genome and annotation files used for the computation of the TSSs. TSS-Captur first filters all TSSs to retain those classified as oTSSs or aTSSs from the MasterTable, extracts the respective downstream genomic region and classifies it as representing either an mRNA or ncRNA. Two different methods are used for this step: CNIT (16), a method based on support vector machines (SVMs), as well as QRNA (15), a comparative sequence analysis requiring a pairwise alignment. Furthermore, the tools TransTermHP (23) and RhoTermPredict (18) are used to predict genome-wide TTSs. While TransTermHP predicts intrinsic terminators via the identification of hairpin loops (23), RhoTermPredict identifies Rho-dependent sites via the identification of Rho utilization (RUT) sites and RNA polymerase (RNAP) pause sites (18). Based on these predictions, a putative TTS (either intrinsic or Rho-dependent) is allocated for each potential transcript. In the case of transcripts classified as ncRNA genes, TSS-Captur computes their secondary structure using RNAFold (24). Furthermore, the putative promoter regions of all transcripts are analyzed using MEME (25) to identify enriched motifs. All results are accessible via an interactive interface.

### Data preparation

After obtaining only orphan and aTSSs from the MasterTable, sequences downstream of these TSSs are extracted from the genome. The mean gene length (μ_G_) is calculated for all entries in the annotation file and used as the transcript length’s upper limit, capped at a maximum of 1000 bp to fulfill the limitations of QRNA. In addition to using μ_G_ as a transcript limit, TSS-Captur also stops transcript extraction if any specified genomic feature is found within the extraction range. Such features include any TSS listed in the MasterTable (either primary, secondary, antisense or orphan) or the start of an entry in the annotation file. By stopping extraction at any TSS, TSS-Captur minimizes the impact of regions related to expressed genes, such as their 5′-UTR regions, on the characterization of the overlooked transcripts. Moreover, this step also prevents the transcripts from overlapping with known but unexpressed genes. Consonant with the recommendation of Rivas and Eddy, all extracted regions <30 nt are removed from the analysis together with their TSS (1,15). After extraction, each transcript is saved in a separate FASTA file, along with its TSS class (oTSS or aTSS). To ensure consistency in reading direction (5′-3′), the complement of reverse strand transcripts is computed.

### Classification of transcripts

For the characterization of transcripts, TSS-Captur uses the comparative genomic tool QRNA and the SVM-based *ab initio* tool CNIT (15,16). Running both classifiers strengthens TSS-Captur’s prediction by accounting for different sequence characteristics and evolutionary aspects of the classification. CNIT requires only the transcript sequence as input and offers two pre-trained models: one trained with animal data and the other with plant data. To our knowledge, no similar tool has been developed for prokaryotes. To ensure the appropriate application of CNIT to prokaryotic data, both models were tested on a subset of annotated genes (see ‘Verification’ and ‘Use Case’ below for details). Unlike CNIT, QRNA relies on a pairwise alignment. To determine the most appropriate alignment for each sequence, TSS-Captur uses BLAST and the nt database (26). A recommended optimal evolutionary distance between sequences, which conserves structure (for ncRNAs) and still returns plausible alignments, is within the range 65–85% (11,15). To achieve such a pairwise alignment, the sensitive discontinuous dc-Megablast search strategy of BLAST is employed, since it mainly returns hits with 80% similarity (27). To address the long runtime and the high sensitivity of this search approach, TSS-Captur reduces the search space in the database to consider only species within the same genus as the analyzed organism. This is achieved by using the organism’s accession code and the ETE toolkit (28) library to query the NCBI Taxonomy. The hits reported by BLAST are evaluated according to the following criteria: (i) the sequence similarity is within the optimal range and (ii) the coverage of the transcript is maximized, prioritizing conservation at the 5′-region of the query (see Supplementary Section SB for details). For each transcript, the best alignment is stored in a FASTA file for usage in QRNA.

The outputs of both tools are compared to compute a final call for each transcript. Both CNIT and QRNA provide a class prediction, a prediction score, and the best functional genomic subregion (e.g., the predicted coding-sequence region for an mRNA transcript). TSS-Captur computes a class-specific *z*-score normalization to enable a comparison of the two methods’ prediction scores. In case of agreement, the output of the best-scoring program is used. In the case of class disagreement of the two tools, TSS-Captur proceeds as follows:

- If either of the tools predicts a coding gene (i.e. mRNA), and the other an ncRNA, the mRNA class is chosen (see ‘Verification’ for details).
- If QRNA returns no prediction due to a missing alignment or due to choosing the null model (*OTH*), CNIT’s prediction is used.

Since both tools also return the coordinates of the most probable functional subsequence, a putative 3′-end for each transcript is computed during this step. However, this is refined by identifying a nearby putative TTS in the following step.

### Transcription termination sites

TSS-Captur makes its termination site prediction in a genome-wide manner using TransTermHP (23) in the case of intrinsic termination and RhoTermPredict (18) for Rho-dependent termination. TransTermHP is run using nocoRNAc (29) as a wrapper, since it is optimized for whole-genome analysis and directly filters terminators with low confidence scores (below 75). Both tools report annotation files (GFF files) with predicted genome-wide termination regions and their corresponding confidence score.

After the aforementioned transcript classification, TSS-Captur associates transcripts with potential terminators. For each transcript, all predicted TTSs starting within the coordinates defined by QRNA or CNIT are considered. Each putative TTS is evaluated by considering its prediction score and its distance to the 3′-end predicted during classification (see Supplementary Section SC for details). The best-scoring TTS is taken for each transcript.

The transcript’s end is then adjusted according to the type of terminator identified. For intrinsic terminators, the end of the hairpin loop marks the TTS. In Rho-dependent cases, the RNAP pause site (150 nt downstream of the RUT site) is analyzed, as it may contain either a hairpin or only a RNAP-pause site (30). If a hairpin structure is detected, both the RUT site and hairpin are included in the termination region, with the TTS placed directly behind the hairpin. If no hairpin is found, the end of the RNAP pause site is considered as the transcript’s end. This process defines a TTS and a transcript length (TTS position−TSS position) for each transcript.

### Secondary structure prediction

For each transcript classified as an ncRNA, RNAFold (31) is used to compute the secondary structure with the minimum free energy. This RNA structure is saved as a JPG file and linked in the interactive report.

### Promoter region analyses

The putative promoter region of each accounted TSS is analyzed using MEME (25,32) to identify over-represented motifs. Such enriched motifs could be either novel regulatory elements or also known transcription factor binding sites, such as the Pribnow-Box or the -35 box. Identifying such motifs can strengthen the validity of the TSS signals. For this analysis, TSS-Captur extracts the 50 bp upstream region of each TSS to be passed to MEME, which identifies a user-defined number of motifs ranging from 5 to 20 bp in length. As long as no >1000 TSSs are analyzed in one batch, MEME will also provide the start of each motif in each sequence, which is essential for identifying common regulation motifs. Results are provided by MEME in a user-friendly HTML report and as an XML file. The XML output is further converted into a TSV file for easier data manipulation. Both formats are included in TSS-Captur’s final output.

### TSS-Captur result interface

To streamline exploration of TSS-Captur’s output, a comprehensive HTML report summarizes the results in one interface (see Supplementary Figure S2), via interactive tables for each characterization step. The ‘overview’ page offers a summary of analyzed and discarded TSSs, including their classification as either orphan or antisense. The central table on this page merges important data: TSS position, strand, predicted class (mRNA/ncRNA), promoter motifs, among other information. Moreover, the page integrates the visualization of the secondary structure for each transcript predicted as ncRNA. The pages on the ‘terminator allocation’ and ‘classification’ provide in-depth information on the corresponding processes. For example, the ‘classification’ page allows users to trace the rationale behind each transcript’s assigned class. The MEMEHTML report is directly integrated in TSS-Captur’s report, allowing a detailed inspection of promoter motifs and leveraging all of MEME’s existing visualization advantages, as well as the other tools from the MEME Suite (e.g., TOMTOM (32)). Finally, the ‘ignored’ *TSS* page lists TSSs excluded by TSS-Captur due to their short length.

## Results

### Verification of TSS-Captur

To assess the overall performance of TSS-Captur, we used the expression data for *Campylobacter jejuni* str. NCTC 11169 published by Dugar *et al.* (4) to compute a MasterTable using TSSpredator, with default parameters. The annotation file (RefSeq ID NC_002163) was used to classify the TSSs with respect to annotated genes. From this set, we selected a subset of 100 primary TSSs (pTSS) and their corresponding annotations, equally balancing both RNA classes (ncRNA and mRNA).

First, we extracted the transcripts using the exact coordinates of each annotated gene of the subset to assess the two models of CNIT (animal- and plant-based) with respect to sensitivity, specificity and precision. Both models demonstrated high sensitivity, specificity and precision (all ≥0.82; see Table 1), indicating that the use of CNIT is suitable for prokaryotes. However, the ‘plant’ model delivered better results (≥0.92; Table 1) and is therefore used subsequently.

**Table 1.** Evaluation of CNIT’s performance on *C. jejuni*’s subset. Each model was evaluated by computing the sensitivity, specificity and precision concerning the annotated RNA class

Model	Type	Sen.	Spec.	Prec.
**Plant**	mRNA	0.92	1	1
	ncRNA	1	0.92	0.926
**Animal**	mRNA	0.82	1	1
	ncRNA	1	0.82	0.847

**Figure 2.**
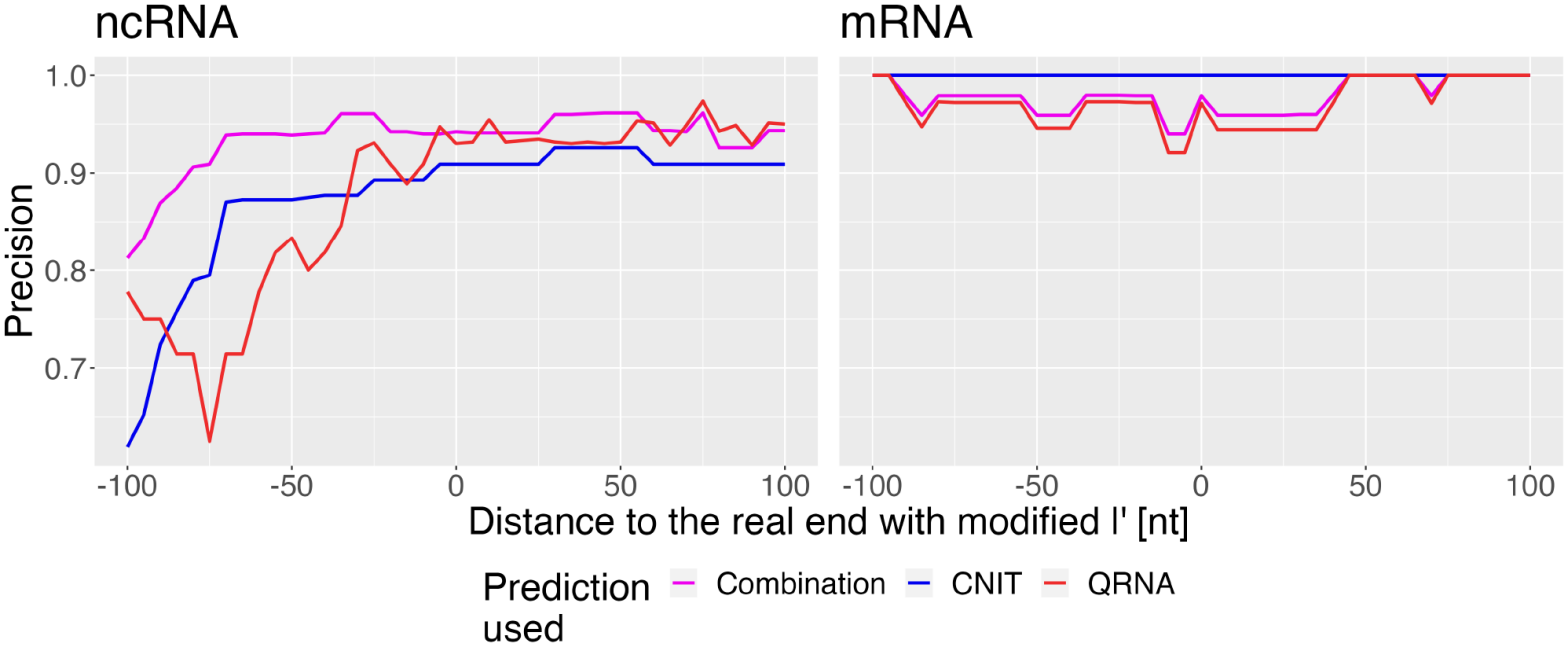
Evaluation of RNA classification. The three different types of predictions are shown: CNIT, QRNA and the combination implemented in TSS-Captur. For each prediction type and for each modified *l*′, the precision was computed for each type of transcript (ncRNA and mRNA).

Afterwards, the influence of the extracted transcript’s length on the overall RNA classification implemented in TSS-Captur was assessed. For each TSS and its annotated transcript end *l*, multiple regions with varying end *l*′ were extracted, with *l*′ = *l* + *t* and *t* = 5*i*, *i* ∈ [ − 20, 20]. To achieve a larger dataset, we lowered the 30 nt extraction threshold to 5 nt. Based on the RNA class (mRNA or ncRNA) from the annotation file, we computed the precision for the three classification approaches (CNIT, QRNA and TSS-Captur’s resulting decision of the combination of both methods). This analysis revealed that by combining both approaches as implemented in TSS-Captur, the highest precision (within the range 0.8–0.95) across most of the transcripts for ncRNA genes was achieved, while CNIT’s precision was the highest for mRNA genes across all lengths (Figure 2). Still, the combination implemented in TSS-Captur returned also precise results for mRNAs (>0.95) across all transcripts’ length.

**Figure 3.**
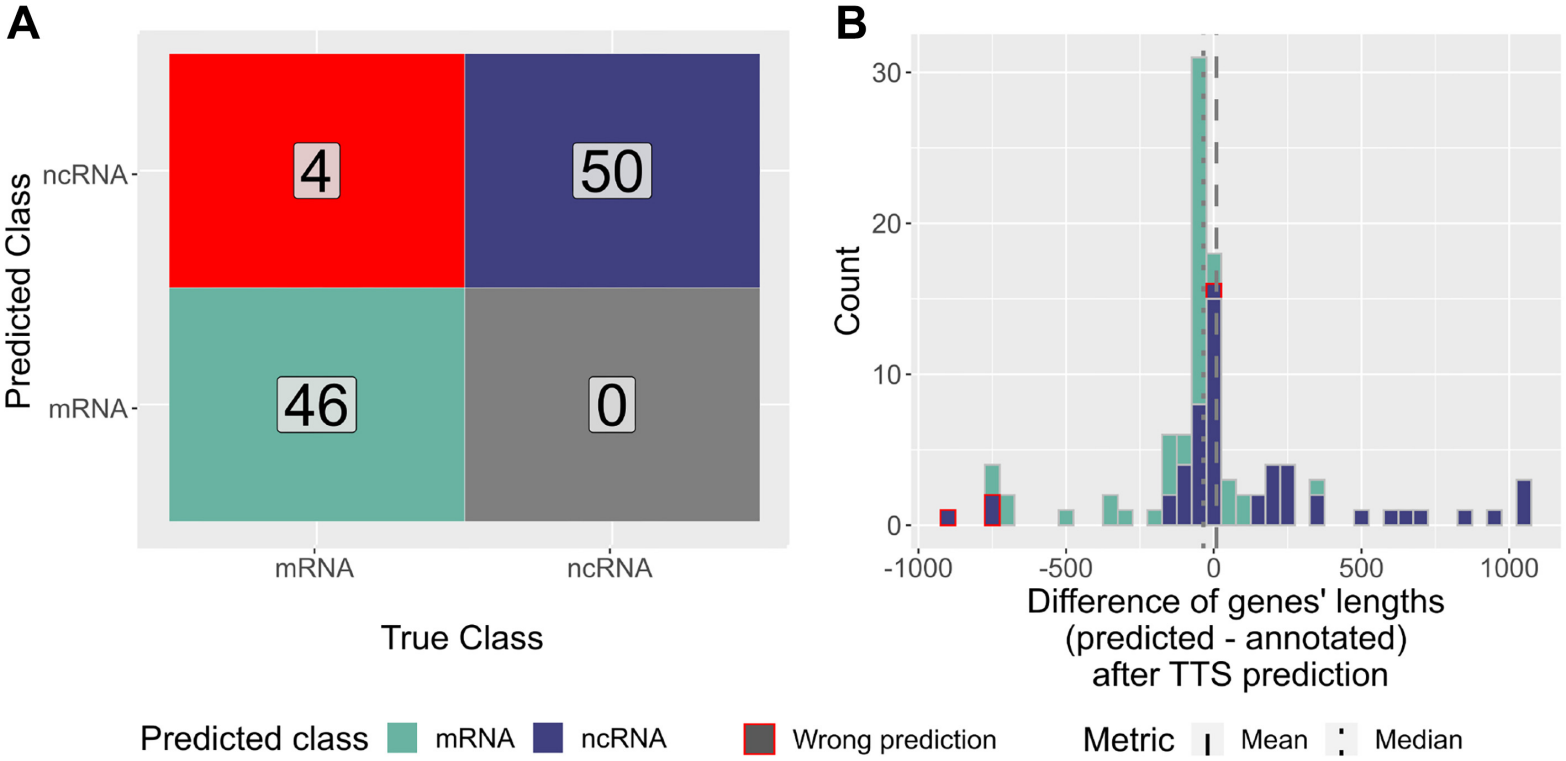
Evaluation of TSS-Captur by treating pTSSs as oTSSs. (**A**) Confusion matrix on the real and predicted RNA classes for the transcripts. (**B**) Distributions of the genes’ differences (predicted—annotated) by predicted RNA class (bin width = 50 nt). The mean and median differences are also shown. The bins containing wrongly predicted RNAs are highlighted.

Lastly, we modified the MasterTable to assess the whole process behind TSS-Captur. We removed all oTSSs or aTSSs from the MasterTable to avoid their analysis in TSS-Captur. The entries in the MasterTable and the annotation file for the 100 pTSSs of the subset were modified to simulate orphan TSSs. The remaining TSSs were left unmodified to have a feasible MasterTable. The output of TSS-Captur was compared with the original classes and lengths. All ncRNA genes were correctly classified, and of the 50 mRNA genes, only four were incorrectly classified as ncRNAs (Figure 3A). For the prediction of the gene’s length, the difference between the predicted and the annotated length was computed, returning a mean difference of 9.2 nt and a median of -35 nt (Figure 3B). Fifty-seven genes had an absolute length difference ≤100 nt, while 74 genes had an absolute length difference ≤250 nt. For those 18 genes with an absolute length difference ≥500 nt, a clear difference in the classes was observed. On the one hand, eight coding genes were strongly underestimated in their length. On the other hand, nine ncRNAs were greatly overestimated in their length.

### Use case: *Streptomyces coelicolor*

To demonstrate the applicability of TSS-Captur, we analyzed a genome-wide TSS dataset of *Streptomyces coelicolor* (33). For this, the expression data were analyzed using TSSpredator with sensitive parameters and an annotation file for the organism (RefSeq ID NC_003888.3). This resulted in a total of 1660 antisense and orphan TSSs that were passed to TSS-Captur as input, together with the genome sequence and annotation file.

**Figure 4.**
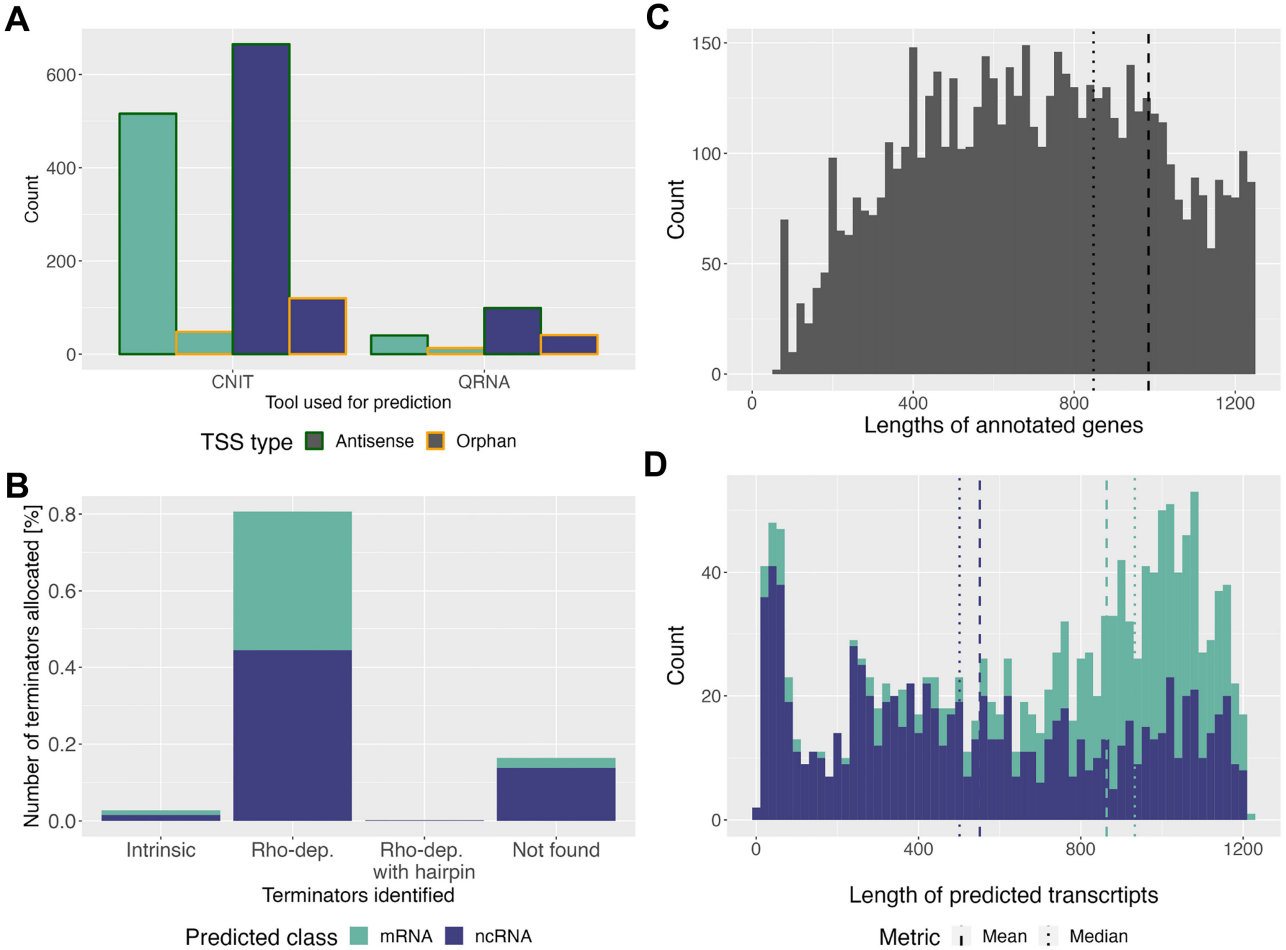
Visual summary of TSS-Captur output for *S. coelicolor*’s data. (**A**) Summary of the predicted RNA classes for each transcript by the tool used for prediction and the original TSS type. (**B**) Distribution of the allocated terminator regions with respect to their type. (**C** and **D**) Histogram (bin width = 20) of the length for annotated genes (C) and for predicted transcripts (D). Only annotated genes <1250 nt are shown for a better comparison with the predictions.

One hundred and eighteen of these TSSs were filtered out, since they would have produced transcripts of a length below our threshold of 30 nt. Of the remaining 1542 TSSs, 925 regions were predicted as ncRNA genes and 617 as coding genes (Figure 4A and Supplementary Figure S2). The majority of the analyzed transcripts was predicted to have a Rho-dependent terminator site ($80.8\%$), $2.8\%$ showed intrinsic termination and for $16.4\%$ of the sequences no terminators were predicted (Figure 4B). The predicted ncRNA transcripts as well as coding genes displayed a wide range of lengths (Figure 4D). With a median length of 933 nt, coding genes are on average around twice as long as ncRNAs, which show a median length of 501 nt. The median and mean gene length predicted for mRNA transcripts is also close to the mean and median length of all annotated genes of *S. coelicolor* (see Figure 4C). The secondary structure of genes predicted as ncRNAs was also computed and included in the report (see Supplementary Figure S3).

**Figure 5.**
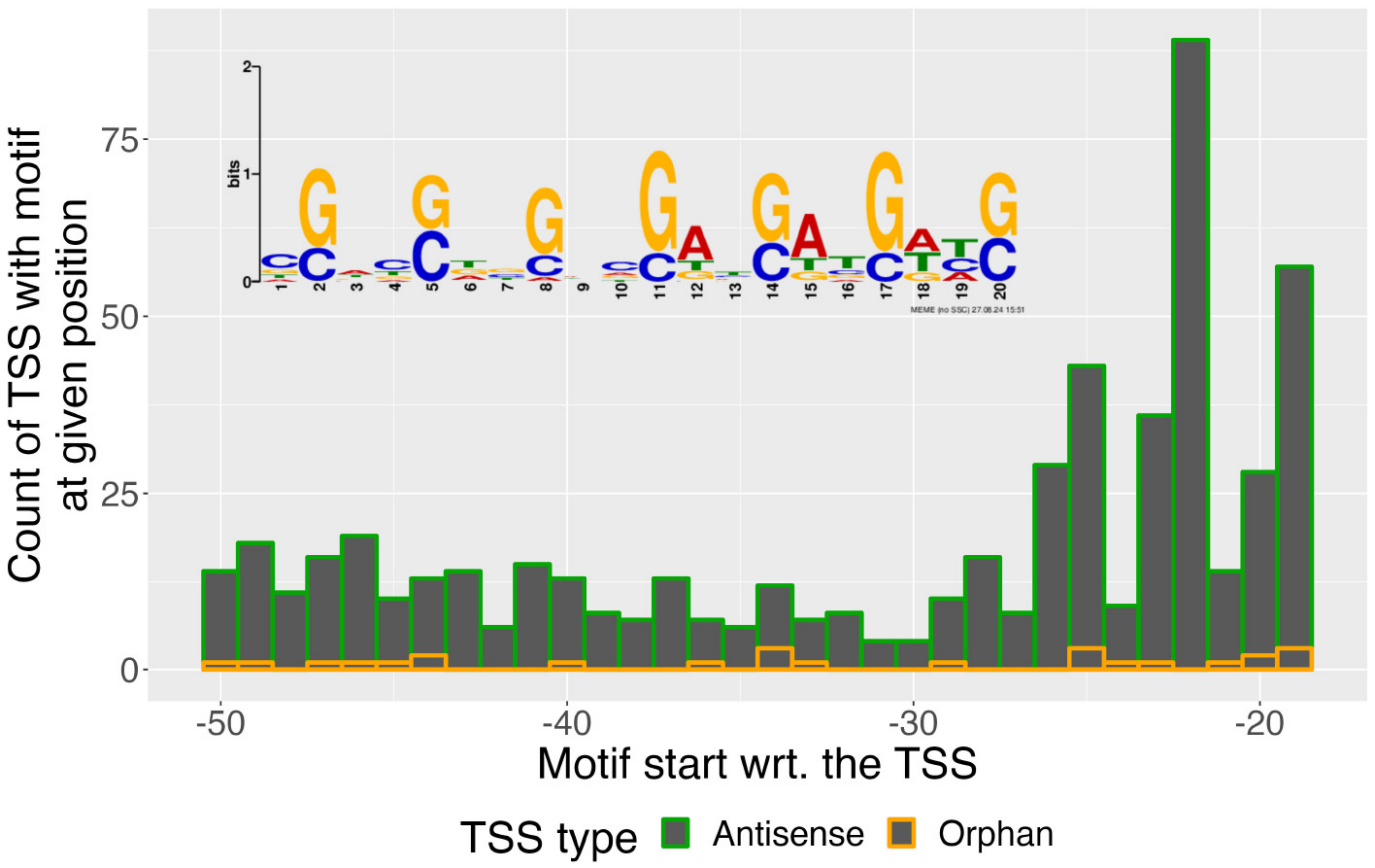
Distribution of start of most enriched motif in the promoter regions for *S. coelicolor*’s analyzed TSSs using MEME.

TSS-Captur identified two enriched motifs within the upstream regions of the analyzed transcripts. The most-significant motif (*E*-value = 2.8 × 10^−469^) was found in 564 promoter sequences, exhibiting a strong G/C pattern and a preference for position 20 nucleotides upstream of the TSS (Figure 5). A database comparison using TOMTOM (34) was started from the report of TSS-Captur. However, it did not return any matches for this motif.

Another study by Vockenhuber *et al.* (35) identified 63 putative small ncRNA genes including their TSSs and TTSs based on RNA-seq. Eleven of them were confirmed using northern blot. We compared the 63 putative ncRNAs as predicted by Vockenhuber *et al.* with the results generated by TSS-Captur based on the study by Jeong *et al.* (33). Since both studies analyzed *S. coelicolor* under different conditions, and the TSS inference methods also differed, the data had to be made comparable. For this, we reduced the sets of TSSs computed with Jeong *et al.*’s data to those that had a distance of ±15 nt to the regions reported by Vockenhuber *et al.* This comparison revealed 23 intersecting TSS positions, including six that had been confirmed using northern blot (Supplementary Table S1). Of all overlapping transcripts, TSS-Captur classified 18 as ncRNA genes, including the six genes confirmed using northern blot. Compared to the lengths reported by the study, TSS-Captur predicted longer transcripts (Supplementary Figure S4). To further validate our predictions, we compared all transcripts classified as ncRNA genes against the RFAM database (36). This returned 11 unique hits (Supplementary Table S2), of which three hits were transcripts already identified in the Vockenhuber *et al.* study.

### Runtime analysis

**Figure 6.**
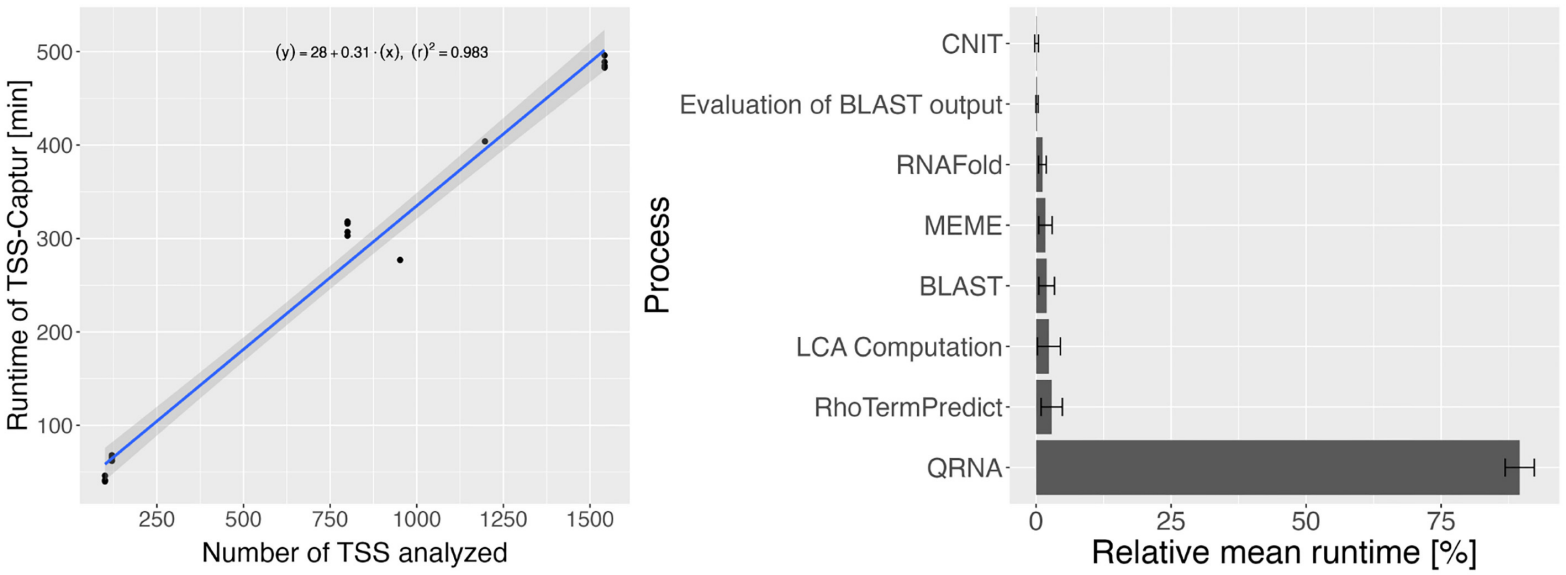
Runtime analysis of TSS-Captur. The runtime of 18 analyses is visualized together with the computed linear regression line (left). The mean of the runtime as well as the standard deviation of each process was computed and shown here in % relative to the overall runtime (right). Processes that were faster than 1 min (e.g., TransTermHP) are not shown.

The runtime of TSS-Captur was assessed using multiple datasets (in total, 18 different runs were conducted) including the conduct of all genome-wide analyses (termination site predictions, promoter identification, etc.). It was performed on a server with an Intel(R) Xeon(R) E5-4610 v2 @ 2.30GHz CPU using at most four cores. The results show a clear linear dependency on the number of TSSs (Figure 6, left) with an increase in the runtime by 18.6 s per TSS. In all runs, the most time-consuming step is the QRNA analysis ($89.5\%$ of total; see Figure 6, right).

## Discussion and outlook

Genome-wide base-resolved transcriptome maps offer insights into regulation regions of annotated genes (e.g., 5′-UTRs, promoters) (5,6). However, they also reveal strong transcription signals in unannotated regions—a rich source of potentially undiscovered genes. Our exploration tool, TSS-Captur, unlocks the potential of these sites, enabling user-friendly analysis that combines genomic and transcriptomic data for their characterization.

The main back-end component of TSS-Captur is a pipeline implemented using Nextflow. Among the advantages of Nextflow are its declarative syntax, as well as modularity and extensibility, which facilitate multiple aspects of the identification and characterization of TSSs.

For the classification of previously uncharacterized transcript sequences, both intrinsic genomic features (CNIT) and evolutionary conservation (QRNA) are considered. Similarly, the TTS prediction combines complementary methods (TransTermHP and RhoTermPredict) for a broad analysis. The analysis of secondary structures and promoter regions offers insights into the function of the transcript as well as its regulation mechanisms. A unified report facilitates exploration of the results.

To validate our approach, we used a dataset based on pTSSs in *Campylobacter jejuni*. We analyzed the precision of the RNA classification across varying transcript lengths, demonstrating that our combined approach provides a balanced performance for both gene classes. By simulating orphan TSSs, we evaluated the overall performance of TSS-Captur. The results showed accurate classification for all ncRNAs and almost all protein-coding genes, while lengths’ predictions vary in accuracy across genes.

Our analysis of the *S. coelicolor* TSS dataset showcases the utility of TSS-Captur in exploring unannotated genomic regions. Of particular interest is the identification of a highly enriched G/C motif upstream of many of the unannotated transcripts. While no direct matches were found using TOMTOM, this motif might be associated with G-quadruplex regulation mechanisms known to operate in *S. coelicolor* (37). This finding demonstrates how TSS-Captur facilitates hypothesis generation. Via the report of TSS-Captur, other tools from the MEME Suite (32) are available to the user to further investigate identified enriched motifs within the promoter regions.

TSS-Captur’s predictions exhibited some overlap with the experimentally validated ncRNAs from Vockenhuber *et al.* (35). Here, it is important to remark that the experimental conditions between the analyzed TSSs with TSS-Captur and the study of Vockenhuber *et al.* differed, hence not all transcripts might be present. This, together with our choice of using the TSS prediction of TSSpredator with the most sensitive parameters, explains the rather low rate of commonly called TSSs. Nonetheless, the confirmation with the study, along with the RFAM validation of some predicted ncRNAs, emphasizes how TSS-Captur can be used to detect novel ncRNAs.

Both TSS-Captur and ANNOgesic use a number of different backend tools for transcript characterization. While ANNOgesic currently offers a larger range of methods than TSS-Captur, addressing additional aspects of transcriptomic data annotation, it also has some limitations. ANNOgesic’s reliance on existing databases and known mechanisms (like the Shine-Dalgarno sequence) for transcript characterization may limit the discovery of novel genes. In contrast, TSS-Captur’s classification relies on more abstract characteristics of the transcripts, with the potential for uncovering previously unknown mechanisms of transcription. Furthermore, by including RhoTermPredict, TSS-Captur accounts for Rho-dependent termination, a well-known mechanism in prokaryotes (30). Moreover, ANNOgesic’s command-line interface and lengthy documentation pose challenges for users lacking extensive computational skills. One motivation to develop TSS-Captur was to provide users with a user-friendly and accessible tool. Our web-interface simplifies file parsing, eliminating the need for inexperienced users to engage in direct command-line interaction. Also, TSS-Captur unifies results within a single interface, offering data exploration alongside convenient access to secondary structure visualizations and external analysis options, such as MEME.

Due to the flexibility of Nextflow, new modules, tools or even other data sources can be easily included in TSS-Captur. One issue that we identified in our two use cases is the prediction of the 3′-end of transcripts. In particular, the lengths of predicted ncRNAs often seem to be overestimated. This was revealed by the validation study, as well as our observation that TSS-Captur’s predicted transcripts often aligned only with subsequences within the RFAM entries. In the future, we may consider integrating more recent tools or other data sources for the prediction of the 3′-end, such as TermNN (38) or Term-seq data (39).

For the classification step of uncharacterized transcript regions, we used two complementary methods and thereby provide a general idea of the transcript’s function (either mRNA or ncRNA). Nonetheless, there remains an opportunity to advance the classification of prokaryotic transcripts. For instance, new methods for intrinsic classification, specifically trained with prokaryotic data, could be developed. These approaches could yield even more precise predictions about transcript classes, though one must account for the fact that sequence homology-based classification typically offers higher precision, especially as the number of sequences increases (11,40). This is particularly true when intrinsic features alone are insufficient for accurate classification, as it is often the case with short-length sORFs (41). Moreover, improvements are possible not only in classification but also in the identification of the most likely secondary structures for ncRNA genes. By considering homologs with similar, though not identical, primary structures, tools like Infernal can incorporate this possibility of structural prediction (42). However, the identification of the relevant set of homologs is not trivial and often requires manual intervention, making such an approach difficult to automate (11).

With respect to the classification, it needs to be acknowledged that TSS-Captur defines two distinctive classes (mRNA and ncRNA) for a transcript. This strict classification might not be adequate for all transcripts, such as dual-function sRNAs (43,44). This lesser known class of genes consists of two components: a small ncRNA and a small open-reading frame component. The limited number of relevant prokaryotic examples hinders the generalization of identification methods for this class of genes (44). Nevertheless, any future development that provides a clearer classification for such transcripts can be easily integrated in TSS-Captur due to the Nextflow-based structure.

In summary, starting from experimental TSS data, TSS-Captur predicts the characterization of unclassified signals and allows a user-friendly exploration to complement prokaryotic annotation tools, contributing to the understanding of bacterial transcriptomes. By combining a user-centric design, TSS-Captur empowers researchers with diverse technical backgrounds to characterize experimentally detected transcription signals and eventually accelerate the discovery of novel genomic elements and their regulatory mechanisms.

## Supplementary Material

lqae168_Supplemental_File

## Data Availability

TSS-Captur can be accessed at https://tsscaptur-tuevis.cs.uni-tuebingen.de/. The source code is available via Zenodo at https://zenodo.org/doi/10.5281/zenodo.12527008. The datasets used for this study are also available online at https://zenodo.org/doi/10.5281/zenodo.12526907. All tools required for the pipeline have been containerized into a Docker image and are publicly available at https://hub.docker.com/r/mwittep/tsscaptur.
