# Supplementary material for: TSS-Captur: a user-friendly pipeline for characterizing unclassified RNA transcripts
Source: NAR Genom Bioinform. 2024 Dec 18;6(4):lqae168. doi: 10.1093/nargab/lqae168 (PMC11655288; doi:10.1093/nargab/lqae168)
Supplement: lqae168_Supplemental_File [file lqae168_supplemental_file.pdf]

Supplementary material for TSS-Captur: A User-Friendly Pipeline for Characterizing Unclassified RNA transcripts

Mathias Witte Paz, Thomas Vogel & Kay Nieselt

A The TSS-Captur pipeline

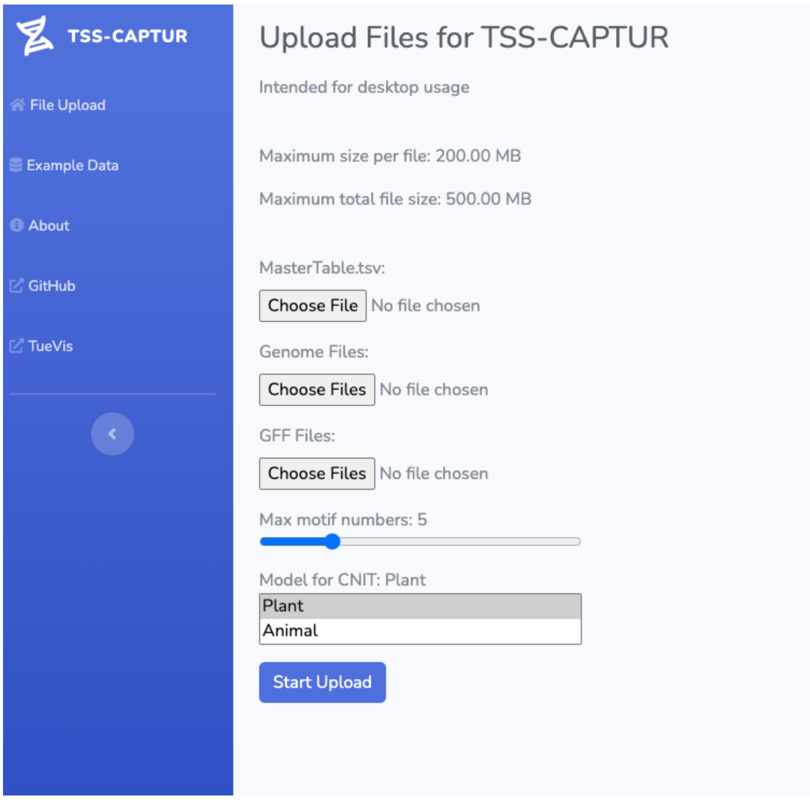

Figure S1: Snapshot of the web-interface of TSS-Captur. It allows the user to upload their files, as well as selecting a few parameters for the pipeline. Two other pages provide more information wrt. TSS-Captur. The *Example Data* page links different datasets that can be used directly in TSS-Captur, as well as their precomputed reports. The *About* page provides an overview of the pipeline, such as the tools used for characterization.

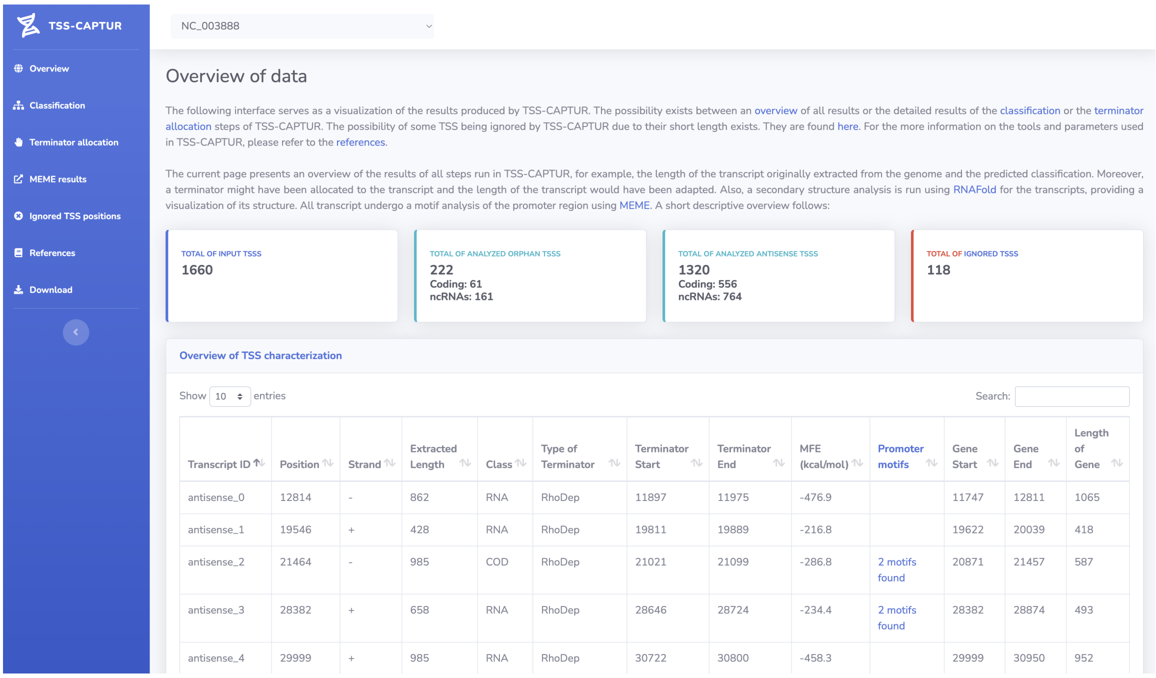

Figure S2: Snapshot of the interactive report of `TSS-Captur` visualizing *S. coelicolor*'s data. The interface provides different levels of details for each analysis step run in `TSS-Captur` that can be accessed via the navigation menu (left). An overview of the data is provided by summarizing main results of the analyzed TSSs. The information regarding each transcript is listed in a tabular view that allows sorting and filtering.

B Evaluation of BLAST's hits

After `BLAST` returns a set of local alignments A for each transcript, the optimal hit H^* must be selected. Instead of relying solely on the best E-value or bit-score, other characteristics are crucial. The ideal pairwise-alignment hit should fulfill the following criteria:

- The distance to the TSS (D_{TSS}) should be minimal.
- The similarity Sim should be within the range of $65 - 85\%$.
- The coverage of the query (Cov_q) in the alignment should be maximized.

To evaluate each hit ($Hi \in A$), the scoring function Eq. 1 is used. `qcovhsp`, `qstart` and `pident` are values returned by `BLAST` that represent the coverage of the query, the start of coordinate of the alignment in the query and the overall identity of the alignment, respectively. A weighted average S combines the three scores with weights 0.45, 0.35, and 0.2, reflecting the relative importance of TSS proximity, similarity, and query coverage. For each transcript, the alignment H^* is taken, with $H^* = \arg\max S(H_i), \forall H_i \in A$, and stored in a `FASTA`-file for usage in `QRNA`.

$$\begin{aligned} D_{TSS}(S_q, t) &= \frac{\min(\max(0, S_q - t), t + 20)}{t + 20} \\ D_{Sim}(I_A) &= \frac{\mathcal{N}_{\mu=75,\sigma=8}(I_A)}{\mathcal{N}_{\mu=75,\sigma=8}(75)} \\ Cov_q(C_q) &= \min(1, \frac{C_q - 50}{25}) \\ S(H_i) &= 0.45 \cdot D_{TSS}(\texttt{qstart}_{H_i}, 10) \\ &+ 0.35 \cdot D_{Sim}(\texttt{pident}_{H_i}) \\ &+ 0.2 \cdot Cov_q(\texttt{qhspcov}_{H_i}) \end{aligned} \tag{Eq. 1}$$

C Evaluation of termination sites

During classification, QRNA and CNIT return the most-probable functional subsequence and hence hint for a first 3'-end of the transcripts. After this, TSS-Captur continues with the allocation of a predicted termination site. For each transcript, any termination regions predicted by RhoTermPredict and TransTermHP starting within the subsequence defined by the two classification tools are considered as putative termination sites. Since the used tools might return false-positives, the predictions are scored to identify the most-plausible termination site. We consider two aspects for this evaluation: the confidence value TTS_{score} returned by the corresponding prediction tool, and the location of the termination region wrt. the coordinates reported by the classification step (T_{start} and T_{end}, respectively, for the start and end of the predicted transcript). Note that T_{start} is the same as the TSS position and T_{end} is the putative end predicted by either QRNA or CNIT. The scores of TransTermHP and RhoTermPredict have different ranges, hence they are normalized, computing TTS'_{score} A weighted average is computed using the normalized score and the distances to the TSS and the $3'$-end of the transcript, as seen in Eq. 2.

$$
\begin{aligned}
D_{start} &= \frac{|TTS_{start} - T_{start}|}{length(T)} \\
D_{end} &= \frac{|TTS_{start} - T_{end}|}{length(T)} \\
score &= \frac{TTS'_{score}}{2} + \frac{D_{start}}{4} + \left(\frac{1 - D_{end}}{4}\right)
\end{aligned} \qquad \text{(Eq. 2)}
$$

D Use case: *S. coelicolor*

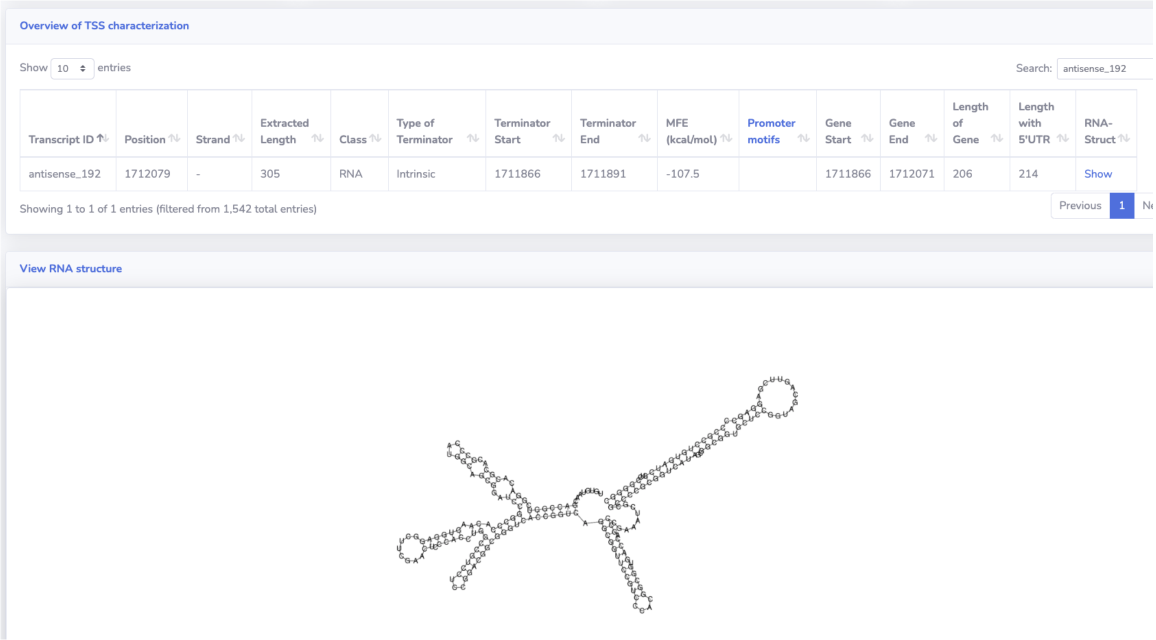

Figure S3: Interactive report of `TSS-Captur` visualizing *S. coelicolor*'s transcript *antisense_192*. The predicted secondary structure is directly visualized in the interface after clicking the corresponding element in the table.

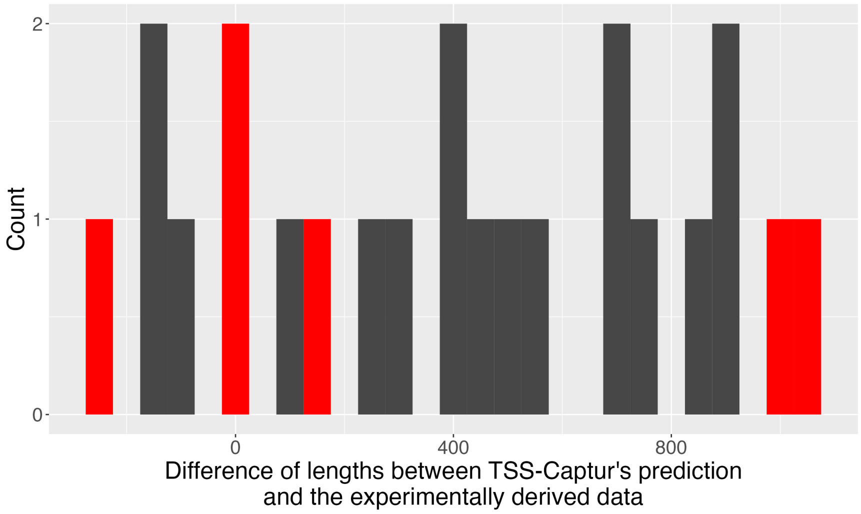

Figure S4: Histogram (bin width = 50) showing the length differences between experimental results presented by Vockenhuber *et al.* [33] and predicted transcripts with `TSS-Captur` for *S. coelicolor*. Highlighted bars (red) represent transcripts confirmed using Northern Blot.

Table S1: Comparison of results of `TSS-Captur` with the study by Vockenhuber *et al.* [33]. The first columns represent the name, start, end and length reported by the study. Transcripts confirmed via Northern Blot (NB) by Vockenhuber *et al.* are indicated. For `TSS-Captur`'s results, the position of the TSS, the start, end, class and length reported by the characterization are shown.

**Study Vockenhuber et al.**					**Results of TSS-Captur**			
**Name**	**Start**	**End**	**Length**	**NB**	**ID**	**TSS**	**Length**	**Class**
scr0991	1045891	1046090	200	Yes	orphan_20	1045891	351	RNA
scr1601	1711967	1712074	214	Yes	antisense_192	1712079	214	RNA
scr1821	1950867	1950948	82	Yes	orphan_32	1950867	1077	RNA
scr2736-1	2982224	2982505	282	Yes	orphan_48	2982224	34	RNA
as2780	3034099	3034209	111	Yes	antisense_376	3034209	1142	RNA
scr3035	3321216	3321353	138	Yes	orphan_59	3321353	140	RNA
as3287	3636488	3636569	82	No	antisense_488	3636567	527	RNA
as3317	3669433	3669657	225	No	antisense_491	3669433	1104	COD
as3321	3673385	3673581	197	No	antisense_494	3673385	709	COD
scr3323	3675098	3675329	232	No	orphan_77	3675098	331	RNA
scr3558	3933527	3933668	142	No	antisense_599	3933677	32	RNA
scr3559	3934693	3934927	235	No	orphan_88	3934687	71	RNA
scr3920	4315358	4315484	127	No	antisense_682	4315355	848	COD
scr3928	4323842	4324023	182	No	orphan_109	4323842	584	RNA
as4261	4674583	4674770	188	No	antisense_744	4674582	36	RNA
as4567	4984635	4984793	159	No	antisense_804	4984793	1059	COD
scr4632	5055055	5055172	118	No	orphan_142	5055173	533	RNA
as4675	5106760	5106941	182	No	antisense_860	5106761	417	RNA
as4699	5124039	5124366	328	No	antisense_870	5124354	621	RNA
as5028	5464169	5464418	250	No	antisense_942	5464419	806	RNA
scr6106	6706584	6706667	84	No	antisense_1148	6706584	932	COD
scr6908	7672451	7672670	220	No	orphan_230	7672451	916	RNA
scr6925	7688336	7688466	131	No	orphan_233	7688336	877	RNA

Table S2: Hits of *S. coelicolor*'s predicted ncRNA genes in the `RFAM` database. The **accession code** column corresponds to the identification used within `RFAM`, while the column **ID** refers to the label given to the transcript by `TSS-Captur`. **Start** and **End** refer to the local coordinates within each transcript that were aligned. The **predicted length** refers to the length predicted by `TSS-Captur`, while the aligned length refers to how much of the transcript was actually aligned. Hits marked with an asterisk (*) represent transcripts that come from the data of Vockenhuber *et al.*.

**ID**	**Target name**	**Acc. code**	**Start**	**End**	***E*-value**	**Pred. length**	**Aligned length**
orphan_87	6C	RF01066	25	100	$1.70 \times 10^{-}05$	167	76
antisense_111	Cobalamin Riboswitch	RF00174	229	331	$4.40 \times 10^{-}14$	330	103
orphan_20	*Cobalamin Riboswitch	RF00174	17	235	$4.90 \times 10^{-}27$	153	219
orphan_233	*Scr6925	RF02826	1	130	$2.10 \times 10^{-}25$	560	130
antisense_192	*Scr1601	RF02830	6	213	$3.00 \times 10^{-}40$	214	208
antisense_840	tRNA	RF00005	401	468	$7.10 \times 10^{-}12$	467	68
orphan_127	ydaO-yuaA	RF00379	182	338	$1.50 \times 10^{-}26$	337	157
orphan_128	Scr4115	RF02829	80	188	$2.40 \times 10^{-}16$	644	109
orphan_157	Scr5239	RF02605	66	224	$3.90 \times 10^{-}32$	543	159
orphan_167	che1	RF02935	370	471	$4.70 \times 10^{-}15$	486	102
orphan_78	Bacterial ssrRNA	RF00177	602	743	$6.80 \times 10^{-}32$	739	142
